# Aberrant perfusion and its connectivity within default mode network of first-episode drug-naïve schizophrenia patients and their unaffected first-degree relatives

**DOI:** 10.1038/s41598-017-14343-7

**Published:** 2017-11-23

**Authors:** Long-Biao Cui, Liu-Xian Wang, Ping Tian, Hua-Ning Wang, Min Cai, Fan Guo, Chen Li, Yu-Jing Wu, Peng-Gang Qiao, Zi-Liang Xu, Lin Liu, Hong He, Wen-Jun Wu, Yi-Bin Xi, Hong Yin

**Affiliations:** 1 Department of Radiology, Xijing Hospital, Fourth Military Medical University, Xi’an, Shaanxi China; 2 School of Medical Psychology, Fourth Military Medical University, Xi’an, Shaanxi China; 3 Department of Psychiatry, Xijing Hospital, Fourth Military Medical University, Xi’an, Shaanxi China; 4 Department of Radiology, Affiliated Hospital of the Academy of Military Medical Sciences, Beijing, China; 5 School of Life Sciences and Technology, Xidian University, Xi’an, Shaanxi China

## Abstract

Neural substrates behind schizophrenia (SZ) and its heritability mediated by brain function are largely unknown. Cerebral blood flow (CBF), as a biomarker of activation in the brain, reflects the neuronal metabolism, and is promisingly used to detect cerebral alteration thereby shedding light on the features of individuals at high genetic risk. We performed a cross-sectional functional magnetic resonance imaging (MRI) study enrolling 45 first-episode drug-naïve patients with SZ, 32 unaffected first-degree relatives of these patients, and 51 healthy controls (HCs). We examined CBF, CBF connectivity, and CBF topological properties. SZ patients showed increased CBF in the left medial superior frontal gyrus and right precuneus compared with HCs, and decreased CBF in the left middle temporal gyrus compared with their relatives. Furthermore, unaffected relatives revealed higher level of CBF pronounced in regions within default mode network (DMN). Both SZ patients and their relatives exhibited dysconnectivity patterns. Notably, as for the network properties, unaffected relatives were with an intermediate level between SZ patients and HCs in the local efficiency and global efficiency. Our findings demonstrate the aberrant CBF of areas within DMN and the CBF connectivity pattern might be a familial feature in the brain of first-episode SZ patients and their relatives.

## Introduction

Schizophrenia (SZ) is a severe psychiatric disease characterized by abnormal mental activities and disturbed behaviors^1^. Common characteristics, such as genetic risk factors, are usually shared among the relatives of SZ patients^2,3^. It has also been shown that family members of SZ patients have more severe subclinical negative symptoms such as social withdrawal and an increased risk for developing SZ compared with relatives of healthy people^4,5^. However, the common and unique functional cerebral deficits between SZ patients and their unaffected relatives remain unclear to a large extent. Elucidation of neural substrates behind SZ is pivotal for treatment of this mental disease.

SZ has been suggested to be associated with alterations in resting-state cerebral blood flow (CBF) in our recent study^6^. With the progress of the noninvasive of arterial spin labeling (ASL) magnetic resonance imaging (MRI), novel and quantitative approach to the measurement of resting-state CBF could be provided. ASL uses magnetically labeled arterial blood water as an endogenous tracer, reflects the level of neuronal activity, and is comparable with positron emission tomography (PET)^7^. Accordingly, SZ patients have exhibited increased or decreased CBF in different brain regions, with hypo-perfusion in the frontal lobes, anterior and medial cingulated gyri and the parietal lobe, while increased perfusion in the cerebellum, brainstem and thalamus, compared with healthy controls (HCs)^8^. Moreover, several resting-state CBF alterations have been related to the core clinical symptoms of SZ^9,10^ or motor behavior^11^. However, the results, especially the activated brain regions, are not all inclusive or consistent^10–12^. A number of confounding factors, such as small sample sizes, heterogeneous treatment conditions, and/or durations of illness may count for the inconsistent results of previous studies.

The CBF may change synchronously in different brain regions to act similar network function. Default mode network (DMN) activates during rest and is deactivated during goal-oriented activity. It comprises the posterior cingulate cortex, precuneus, inferior parietal cortex, medial prefrontal cortex, and medial temporal lobe. Because of the functional connection between the DMN and internal mentation, the activation of DMN might be involved in misattribution of thoughts in SZ. With the boundary between imagined scenarios and blurred external attention, psychopathological phenomena such as auditory hallucinations may thus occur. Kindler *et al*. proved increased CBF connectivity within the DMN in SZ patients^13^. CBF alterations in other brain areas, including the left thalamus, right medial superior frontal gyrus, left insula and postcentral gyrus, were also observed^10^.

Many studies have been done for the individuals at high risk of SZ, including structural changes and functional characteristics. It has been revealed the middle temporal, frontal, cingulate and occipital lobes with thinned cortices in first-degree relatives of SZ patients^14,15^. Moreover, several previous studies reported different patterns of grey matter volume abnormalities in first-degree relatives, although the results remain inconclusive^16–18^. In addition, Goghari *et al*. reported that increased fractional anisotropy in the right fimbria of the fornix for relatives compared to patients and HCs^19^. It has also been suggested several brain regions involving the dorsolateral prefrontal cortex, anterior cingulate cortex, caudate, and hippocampus with functional alterations^20–22^. With the whole-brain functional connectivity analysis, Lui *et al*. showed abnormal connectivity between the thalamus and bilateral parahippocampal gyri^23^. Meanwhile, another study performed by Stolz *et al*. found distributed functional activity in the increased blood oxygenation level dependent (BOLD) response to retrieval in the prefrontal regions, thalamus and insula under task mission^24^. However, ASL provides quantitative measure of perfusion while BOLD techniques represented semi-quantitative evaluation of local oxygenation. Nevertheless, the CBF study comparing the SZ and their first-degree relatives is still lacking.

The present study gathered a dataset in first-episode SZ patients, their unaffected first-degree relatives and matched HCs. Then, pulsed ASL (pASL) technique was adopted. The aim of the current study was to detect the CBF alteration patterns in SZ and their first-degree relatives. The association between CBF alterations and clinical parameters was also evaluated. Finally whether regions with altered CBF have abnormal patterns of CBF connectivity with other brain areas was determined.

## Methods

### Participants

This study was approved by the ethical committee of Xijing Hospital, and all participants provided written informed consent after complete description of the study. All experiments were performed in accordance with relevant guidelines and regulations. A total of demographically matched 51 first-episode drug-naïve SZ patients, 33 unaffected first-degree relatives of these SZ patients, and 53 HCs were included in the experiment. In the subsequent analysis, six patients, one relative, and two HCs with excessive head motion (>3 mm translation and/or >3.0° rotation) were detected and then excluded from the current study. Subjects from our inpatient department or outpatient clinic were assessed according to the Diagnostic and Statistical Manual of Mental Disorders, Fourth Edition, Text Revision (DSM-IV-TR) criteria and consensus diagnoses of SZ were made using all the available information. This was based on the scores of the Positive And Negative Syndrome Scale (PANSS) (score ≥ 60) at the time of scanning^25^. The following exclusion criteria applied to all groups: history of significant neurological or systematic illness, diagnosis of substance abuse in the prior 30 days or substance dependence in the prior 6 months, another axis I or II psychiatric disorder, receiving antipsychotics and/or intervention using transcranial direct current stimulation, transcranial magnetic stimulation, electroconvulsive therapy or behavioral therapy, and pregnancy or MRI contraindications. The Prodromal Questionnaire was used to confirm the absence of any psychotic syndromes in relatives and HCs^26^.

### Image acquisition

As described previously^27–29^, scanning was performed on a Siemens (Erlangen, Germany) 3.0 T Trio MR scanner using the body coil for transmission and an eight channel head coil for reception. A 10-min magnetization-prepared, rapid acquisition gradient echo (MPRAGE: 192 slices, voxel size = 1 × 1 × 1 mm^3^, matrix = 256 × 256, slice thickness = 1 mm, gap = 0 mm, FOV = 256 × 256, repetition time = 2530 ms, echo time = 3.5 ms) image was first acquired. The resting-state perfusion imaging was performed using a pASL sequence (repetition time = 2805 ms, echo time = 13 ms, post-label delay = 1800 ms, flip angle = 90°; field of view = 256 mm × 256 mm; reconstruction matrix = 256 × 256; slice thickness = 3 mm, gap = 0.75 mm; 31 axial slices). The acquisition time was 4 min and 20 s for the resting state ASL scan. During the ASL scan, all subjects were instructed to keep their eyes closed, relax and move as little as possible, think of nothing in particular, and not fall asleep. In order to avoid excessive head motion, we used a custom-built head cushion, reducing motion artifacts during acquisition. Thereafter, subjects with excessive head motion (>3 mm translation and/or 3.0° rotation during scanning, as mentioned above) were excluded from the current study in order to control its potential confound effect^30,31^. Additionally, we carefully checked images of each subject before further analysis, thereby ensuring the quality of data.

### CBF calculation

As we performed recently^6^, data analysis was implemented in Arterial Spin Labeling Perfusion MRI Signal Processing Toolbox (ASLtbx; http://cfn.upenn.edu/~wangze/ASLtbx.php) in accordance with reports by Wang *et al*.^32–34^. The detailed procedures, partly based on SPM12 (http://www.fil.ion.ucl.ac.uk/spm/), included image reorientation, motion correction, coregistering to each subject’s anatomical image then to PET-perfusion templates and finally to Montreal Neurological Institute (MNI) space by means of Affine transformation, spatial smoothing using a 6 mm full-width at half-maximum (FWHM) Gaussian kernel, removing non-brain tissue, as well as CBF quantification.

### CBF connectivity analysis

CBF matrix computation, connectivity matrix construction, and graph analysis were performed in the experiment using the method by Melie-Garcia *et al*.^35^, in which a full description of this approach is available. First, parcellation, with 90 anatomical structures using the automated anatomical labeling (AAL) atlas^36^, was conducted to the preceding preprocessed CBF images for all the participants, yielding a matrix with “n” (number of participants) rows by 90 columns. Second, we defined a connection as statistical associations in CBF between brain regions for a particular parcellation and then constructed CBF connectivity matrix. We also calculated the sparsity/density of these matrices. The sparsity or density of a network is a fraction of the number of edges to the possible number of edges when the network is fully connected. Finally, we estimated global network properties, including clustering index, characteristic path length, and local and global efficiencies, to characterize brain CBF network.

### Statistical analysis

The group differences in CBF were compared in a voxel-wise manner using a one-way analysis of variance (ANOVA) and two-sample *t*-test. Multiple comparisons were corrected using an AlphaSim correction with a threshold of *P* < 0.005 (cluster > 27) according to the Correction Thresholds by AlphaSim module of Resting-State fMRI Data Analysis Toolkit V1.8 (http://www.restfmri.net/forum/REST_V1.8). For two-sample *t*-tests, we used a higher level of threshold, i.e., *P* < 0.005/3 (AlphaSim correction, cluster > 27), due to comparisons for three times. For each subject, Pearson correlation coefficients between brain function measures of each cluster with a significant group difference and the severity of symptoms (PANSS scores) were separately computed for SZ patient group. Correlation analyses were performed with SPSS software (version 13.0, SPSS, Inc.). Significance was set at *P* < 0.05. For CBF connectivity, Fisher’s *z* test was then used to compare correlations between two groups (http://www.fon.hum.uva.nl/Service/Statistics/Two_Correlations.html).

## Results

### Demographic and clinical data

The demographic and clinical data are shown in Table 1. There were significant differences in education years between SZ patients and unaffected relatives, SZ patients and HCs. No other obvious difference was found.

**Table 1:** Demographical and clinical characteristics of three groups.

Characteristics	SZ patients (n = 45)	Unaffected relatives (n = 32)	HCs (n = 51)
Age (years)	26 ± 6	28 ± 5	27 ± 4
Sex (male/female)	25/20	23/9	32/19
Ethnicity	Han (Chinese)	Han (Chinese)	Han (Chinese)
Education (years)	13 ± 2*^,#^	14 ± 2	15 ± 2
Handedness (right/left)	45/0	32/0	51/0
Smoking status (smoker/nonsmoker)	11/34	8/24	18/33
PANSS Positive Score	24 ± 8	—	—
PANSS Negative Score	23 ± 7	—	—
PANSS General Psychopathology Score	49 ± 8	—	—
PANSS Total Score	96 ± 18	—	—

^*^ *P* < 0.05 versus unaffected relatives; ^#^ *P* < 0.05 versus HCs.

### Brain regions with significant differences in CBF

**Figure 1:**
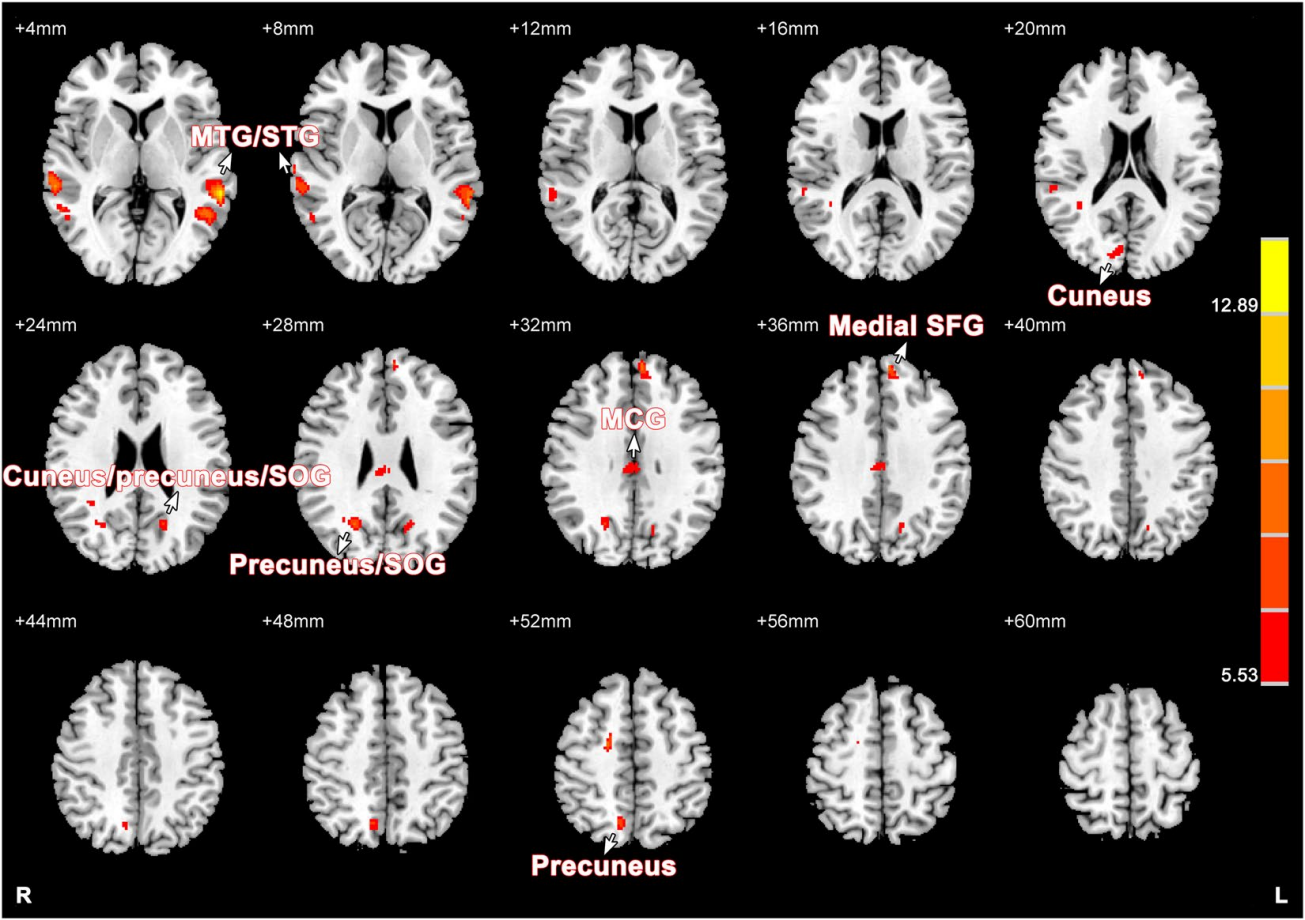
Comparisons of CBF among SZ patients, their unaffected relatives and HCs using ANOVA. The coordinate of each slice is marked in the upper-left. The color bar on the right indicates the F values. Brain regions with difference in CBF are marked by white arrows. MCG, middle cingulate gyrus; MTG, middle temporal gyrus; SFG, superior frontal gyrus; SOG, superior occipital gyrus; STG, superior temporal gyrus.

Whole brain CBF values of each subject were calculated before analysis. Differences between SZ patients and HCs, SZ patients and unaffected relatives, and unaffected relatives and HCs are displayed in Table 2 and Fig. 1. ANOVA showed significant difference of CBF in the bilateral middle/superior temporal gyri, precuneus, cuneus, superior occipital gyri and middle cingulate gyri, and left medial superior frontal gyrus (*P* < 0.005, AlphaSim correction, cluster >27). Compared with HCs, SZ patients had higher CBF values in the left medial superior frontal gyrus and right precuneus (*P* < 0.005, AlphaSim correction, cluster > 27). Analysis between SZ patients and unaffected relatives demonstrated that SZ patients had obvious decreased CBF values in the left middle temporal gyrus (*P* < 0.005, AlphaSim correction, cluster > 27). In addition to the former analysis, we also compared CBF values between unaffected relatives and HCs. Unaffected relatives demonstrated increased CBF values in the bilateral middle temporal gyri and precuneus/cuneus, left superior occipital gyrus, and right superior temporal gyrus (*P* < 0.005, AlphaSim correction, cluster > 27).

**Table 2:** Brain regions with significant differences in CBF of three groups.

Comparisons	Regions	Cluster size	Peak F/T value	x	y	z
ANOVA						
	Left MTG	104	9.8593	−52	−54	2
	Left MTG/STG	175	14.3602	−60	−40	6
	Right MTG/STG	233	10.4084	62	−34	4
	Left cuneus/precuneus/SOG	71	7.2865	−18	−64	24
	Right cuneus	29	6.3264	14	−86	18
	Right precuneus/SOG	72	8.7688	24	−64	28
	Right precuneus	64	7.9461	12	−66	50
	Bilateral MCG	95	6.7861	2	−24	30
	Left medial SFG	88	10.0719	−6	50	34
SZ > HCs						
	Left medial SFG	49	3.9741	−6	52	34
	Right precuneus	37	3.7397	12	−66	52
SZ < Unaffected relatives						
	Left MTG	51	−4.2625	−64	−42	4
Unaffected relatives > HCs						
	Right STG	163	4.5259	66	−32	6
	Right MTG	34	3.6221	54	−58	6
	Left MTG	239	5.3947	−60	−42	6
	Right precuneus	31	3.9009	8	−46	62
	Left precuneus	37	3.8766	2	−66	26
	Right cuneus	59	3.9966	22	−64	28
	Left SOG/cuneus	44	4.0131	−20	−64	24

MCG, middle cingulate gyrus; MTG, middle temporal gyrus; SFG, superior frontal gyrus; SOG, superior occipital gyrus; STG, superior temporal gyrus.

### Correlation analysis

Correlation analysis between image measures and PANSS scores showed that CBF values in the left middle temporal gyrus manifested a significantly negative correlation with PANSS total score (*r* = −0.308, *P* = 0.040). Other results are shown in Table 3.

**Table 3:** Correlation analysis between image measures and PANSS score in SZ patients.

PANSS scores	Regions	*r* values	*P* values
Total score	Left medial SFG	−0.063	0.683
	Right precuneus	0.184	0.227
	**Left MTG**	**−0.308**	**0.040**
Positive score	Left medial SFG	−0.034	0.826
	Right precuneus	0.071	0.643
	Left MTG	−0.271	0.072
Negative score	Left medial SFG	0.064	0.678
	Right precuneus	0.136	0.373
	Left MTG	−0.235	0.119
General Psychopathology score	Left medial SFG	−0.156	0.306
	Right precuneus	0.208	0.170
	Left MTG	−0.201	0.185

MTG, middle temporal gyrus; SFG, superior frontal gyrus.

### CBF connectivity

**Figure 2:**
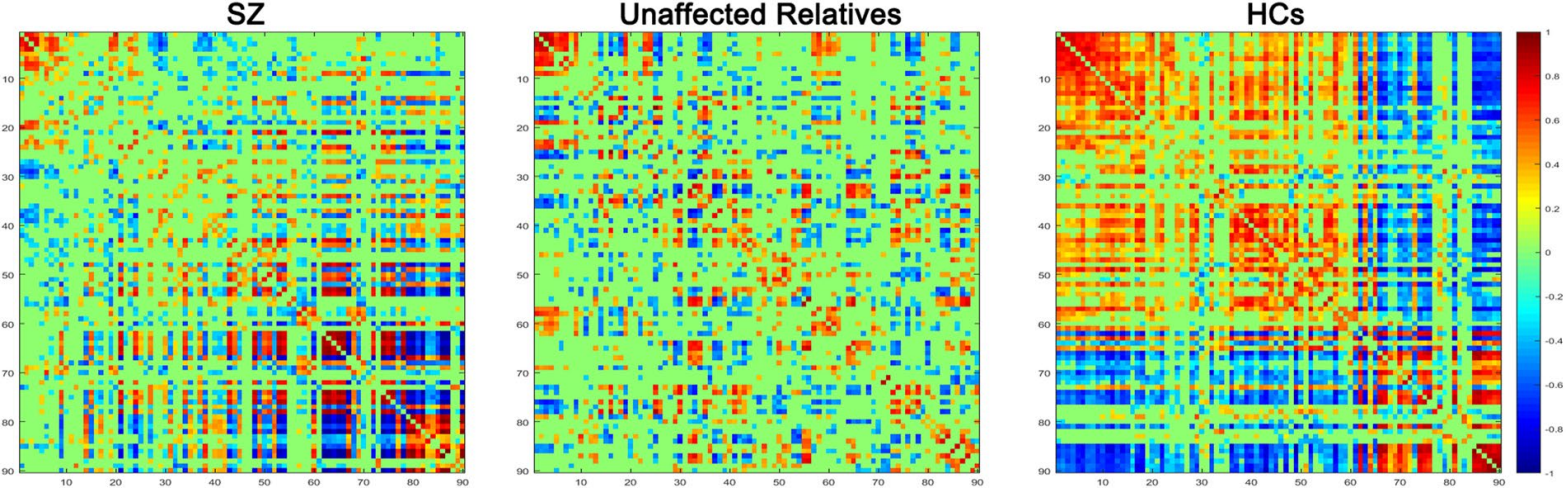
The CBF connectivity matrix obtained by calculating Pearson’s correlation between regional CBF across subjects. Numbers on the left and lower sides of each matrix refer to the corresponding brain regions in AAL template. The color bar indicates the Pearson correlation coefficients.

By calculating Pearson’s correlation between regional CBF across subjects, we obtained three 90 × 90 CBF connectivity matrices, exhibiting correlation coefficient value for each group, as shown in Fig. 2. In each matrix, the green color stand for non-correlation, and red and blue colors represent positive and negative trends of correlation, respectively. All 90 components’ CBF connectivity of whole brain can be observed from the matrix of each group. The sparsity/density of each connectivity matrix was 0.4150 (SZ), 0.3036 (relatives) and 0.6015 (HCs). Similar pattern of CBF connectivity, disconnection, was hence detected in SZ patients and their unaffected relatives.

**Figure 3:**
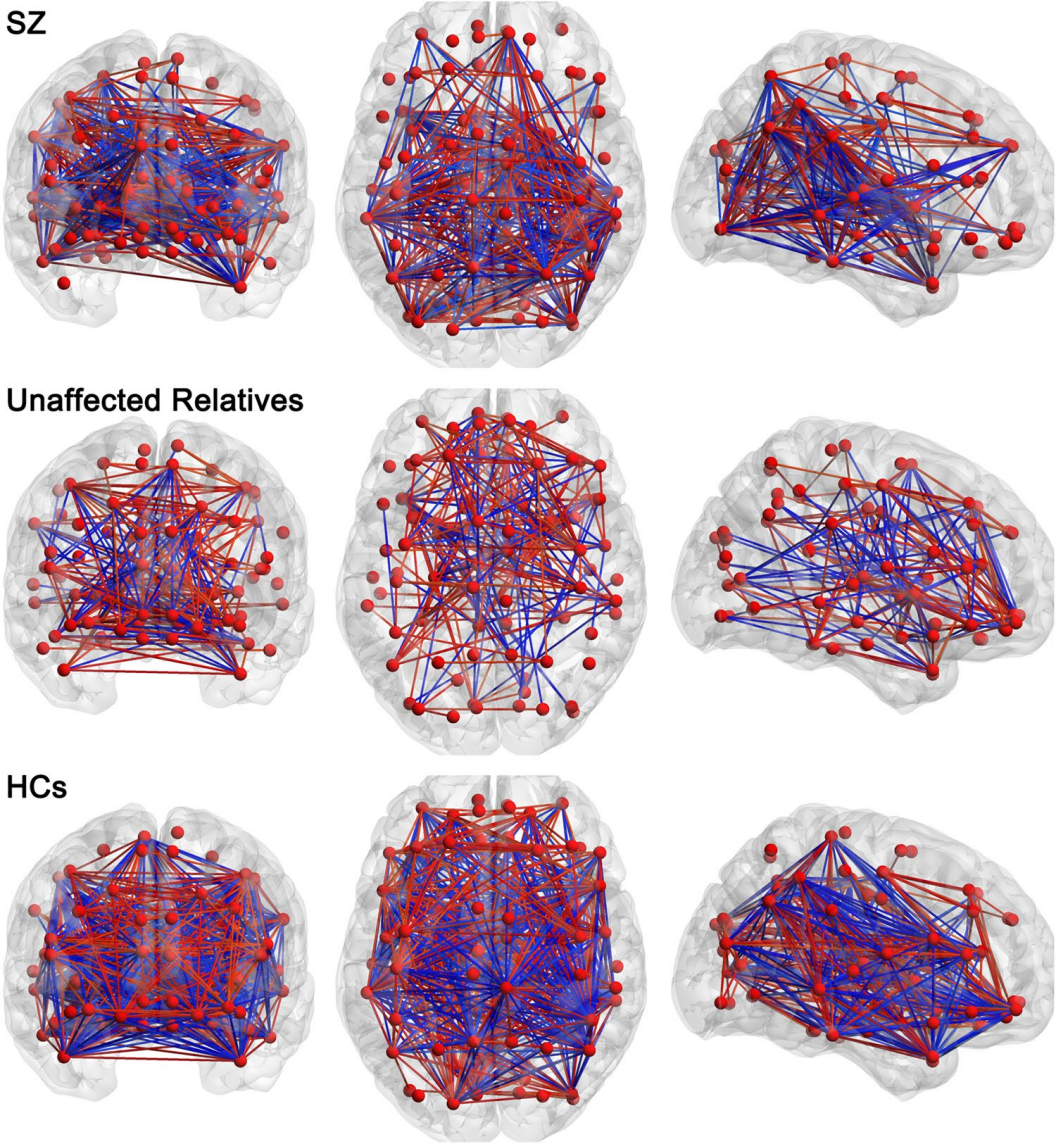
The CBF networks showing connections with correlation coefficient value larger than 0.6 or less than −0.6. Connectivity pattern of each group is in line with the matrix in Fig. 2. Red balls represent the 90 brain regions in AAL atlas. Line color indicates the positive (red) or negative (blue) coefficient.

In order to directly perceive the CBF correlation of each group, we used BrainNet Viewer to present the connectivity patterns with correlation coefficient value larger than 0.6 or less than −0.6, including the connectivity of all 90 modules of the whole brain (Fig. 3). Then, *z* values of connections with significant difference between SZ patients and their unaffected relatives are listed in Table 4, and the complete results are shown in the supplement.

**Table 4:** *z* values of significant connectivity of regions with different CBF between SZ patients and their unaffected relatives.

Comparisons	Connectivity	*z* values	*P* values
SZ > Relatives			
	Left MTG-right hippocampus	3.2682	0.0038
	Left MTG-left auditory cortex	3.2902	0.0036
	Left MTG-right auditory cortex	3.7759	0.0006
	Left MTG-left STG	2.8149	0.0152
	Left MTG-right STG	3.5689	0.0014
	Left MTG-left MTG (temporal pole)	3.3776	0.0027
	Left MTG-right ITG	3.6094	0.0012
SZ < Relatives			
	Left MTG-left lingual gyrus	−3.1136	0.0063
	Left MTG-left IOG	−3.2017	0.0047
	Left MTG-right IOG	−2.9308	0.0109
	Left MTG-right inferior parietal lobule	−3.6329	0.0011
	Left MTG-right SMG	−2.9428	0.0105
	Left MTG-left angular gyrus	−5.3643	0.0000
	Left MTG-right angular gyrus	−2.9255	0.0111
	Left MTG-right putamen	−2.9335	0.0108
	Left MTG-right pallidum	−4.6493	0.0000
	Left MTG-right thalamus	−2.9342	0.0108

IOG, inferior occipital gyrus; ITG, inferior temporal gyrus; MTG, middle temporal gyrus; SMG, supramarginal gyrus; STG, superior temporal gyrus.

**Figure 4:**
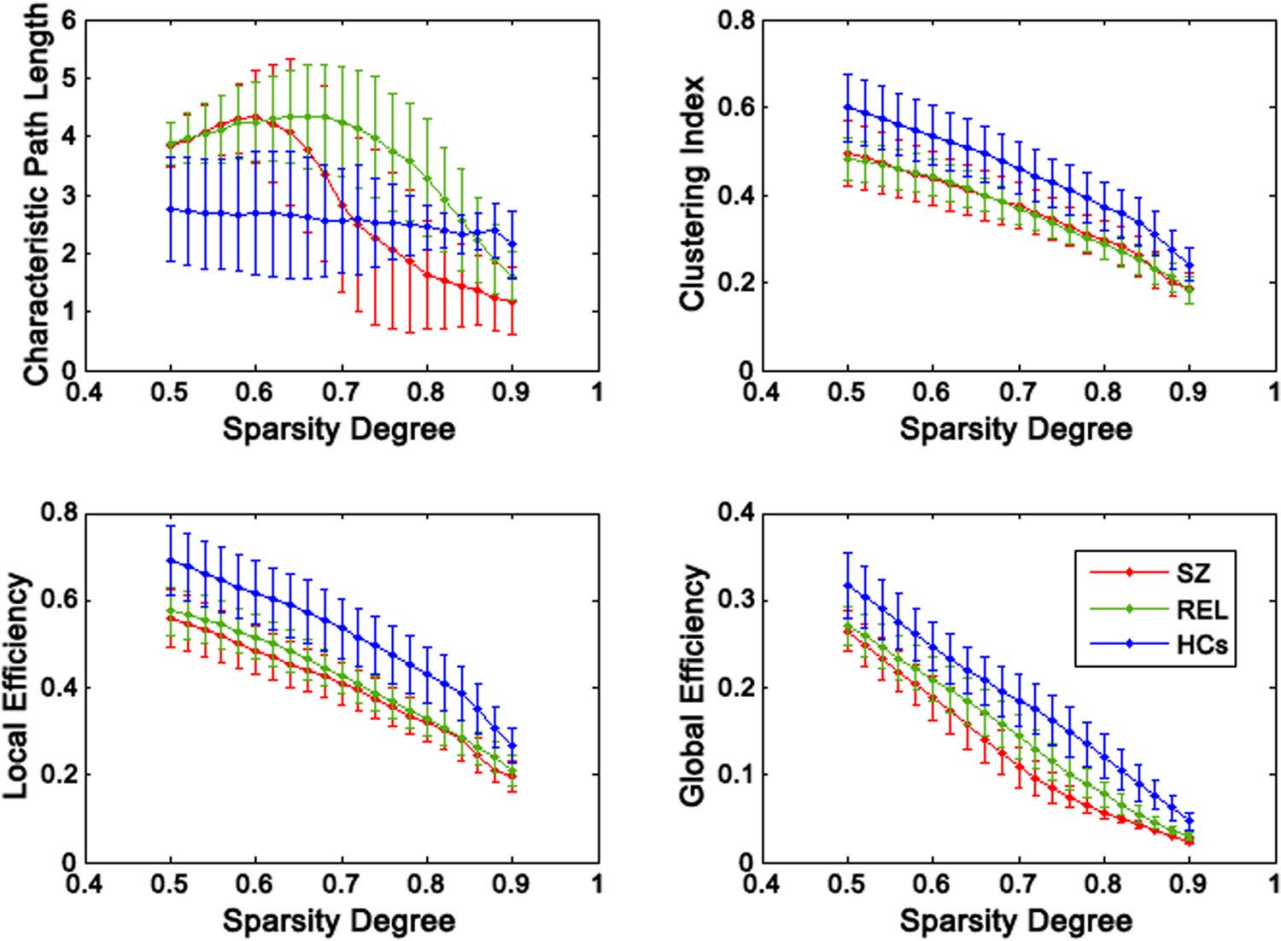
Global network properties as function of sparsity degree. The characteristic path length, clustering index, local efficiency, and global efficiency decrease as the sparsity degree increases. The error bar represents standard deviation. SZ, schizophrenia (red line); REL, relatives (green line); HCs, healthy controls (blue line).

SZ patients and their unaffected relatives showed disrupted pattern of characteristic path length, and they also exhibited relatively small clustering index, and low local efficiency and global efficiency in contrast to HCs (Fig. 4). Moreover, unaffected relatives were with intermediate level between HCs and SZ patients for local efficiency and global efficiency. Generally, both SZ patient group and their unaffected relatives showed deficit communication within the network compared with HCs.

## Discussion

In this study, we used pASL to analyze CBF values and CBF connectivity of each group by recruiting first-episode, drug-naïve SZ patients, their unaffected relatives, and matched HCs, aiming at figuring out similarity between SZ patients and their unaffected relatives, together with differences between the former two groups and HCs. We found that SZ patients exhibited increased CBF values in the left medial superior frontal gyrus and right precuneus as compared with HCs, and decreased CBF value in the left middle temporal gyrus in contrast to their unaffected relatives. CBF value in the left middle temporal gyrus was negatively correlated with PANSS total score. We obtained three 90 × 90 CBF connectivity matrices and the BrainNet Viewer showed the connectivity patterns of each group, from which we can figure the correlation of brain regions.

Lots of scientists focused on functional mechanism underlying SZ and found many enlightening outcomes. But researches using first-episode, drug-naïve SZ patients remain being in demand for the original pathogenesis information because they offer implications without the effect of medicine or stage of disease. ASL is a reliable way assessing the CBF, which utilizes a specific magnetic resonance sequence to quantitatively obtain CBF values noninvasively^37^. In this study, we applied this technique for obtaining the whole brain CBF values in first-episode, drug-naïve patients, their unaffected relatives, and HCs.

Compared with HCs, we found SZ patients had higher CBF values in the left medial superior frontal gyrus, which was consistent with former studies^38,39^. However, some research reported hypo-perfusion in this area^10,40^. The medial prefrontal cortex is regarded as associating with the function of coordinating dynamic interaction between emotional and cognitive signals of the brain^41^, so hyper-perfusion in left medial superior frontal gyrus may lead to some behavioral deficits in SZ patients. In the comparison of CBF values in SZ patients and HCs, we also found hyper-perfusion in the right precuneus in SZ patients, consistent with former research^8^. As we learn from other studies, most demonstrated hypo-perfusion in focal regions, we supposed this difference maybe related with the exclusion criteria and medical background.

Our previous studies indicate abnormalities in DMN^29^ and inter-hemispheric connectivity and reduced volume of several DMN regions in first-episode SZ patients^42,43^. Most recently, we found altered effective connectivity related to a part of DMN, medial prefrontal cortex, in SZ patients using spectral dynamic causal modeling, indicating hippocampal-dorsolateral prefrontal-medial prefrontal hypoconnectivity^27^. DMN, as one of the most acknowledged networks, is related with construction of an integrated self-representation which underlying pathogenesis in SZ^44^. In addition to the left medial superior frontal gyrus, the right precuneus detected in our findings is another part of DMN. It has been reported that in first-episode, drug-naïve SZ patients, several dysconnectivity in DMN and between DMN with other networks could be found^45^. During cognitively demanding tasks, DMN usually tends to represent deactivation, for the purpose of regulating the balance with the central-executive network^46^. While increased CBF values may stand for aberrant activation of DMN in our study, which may cause troubles like failure in constructing proper self-referential stimulations^44^. The left medial superior frontal gyrus, as a part consisted in DMN and left dorsal attention network, plays a crucial role in SZ. Its impaired connectivity with other regions may lead to deficits such as error in processing internal signals^47^, and generating attention signals of current task^48^, impairments in activation of sensory and motor regions^49,50^, and all those alterations may lead to symptoms in SZ. Precuneus, being implicated with perceiving internal and external environment information, processing emotional salient stimuli and working memory dysfunction^51^, was found to be hyper-perfusion in our study. It has been reported that precuneus manifested an increased connectivity within DMN in SZ, in addition to the increased CBF value in this area we found, representing deactivation of DMN during resting state, which may induce defective self-monitoring and other self-centered symptoms in SZ^13^.

Furthermore, Kindler *et al*. reported CBF value in the precuneus was significantly correlated with PANSS score^13^. Although CBF difference in the right precuneus was detected between SZ patients and HCs, we did not observe this correlation in our study. Treatment and sample size, as confounding factors mentioned above, may count for inconsistent results between these two studies. For one thing, a follow-up functional MRI study observed the longitudinal changes in resting-state cerebral activity in first-episode SZ patients^52^. All patients had medications in the study by Kindler *et al*.^13^, but all participants were drug-naïve in the present study. For another, as compared with their study, we had a larger sample size in the current study, which might also have an impact on the statistical analysis. As shown in the correlation analysis, however, CBF value in the left middle temporal gyrus was significantly correlated with PANSS total score, suggesting CBF value in this area could become a potential biomarker in evaluating symptoms in SZ.

As for the difference of CBF values between SZ patients’ unaffected relatives and HCs, we detected higher perfusion predominantly in the regions within DMN in relatives of SZ patients, namely middle/superior temporal gyri, precuneus, and cuneus. Brain structural deficits in twins discordant for SZ were more pronounced in monozygotic than in dizygotic twins^53–55^, suggesting association of cerebral abnormalities with genetic factors for SZ. During the past five years, many structural MRI studies have revealed that gray matter and white matter in individuals at high risk of SZ are unlike controls, but usually to a lesser extent than that of patients, indicating that structural abnormalities may form markers of susceptibility and transition to SZ^56^, despite not definitely consistent findings. Also, a series of studies have demonstrated functional alterations in relatives of SZ patients at resting^20,21,57–60^ or task state^24,61–63^, with significant results found in several specific brain regions. It has been well established that familial risk plays a significant role in the etiology of SZ through family, adoption, twin, and sibling studies. SZ as a hereditary component affects 0.3% to 0.7% of the general population globally according to American Psychiatric Association^64^, whereas first-degree relatives have increased risk of developing SZ, with an actual prevalence of approximate 10%^2^. In genetic epidemiology studies, first-degree relatives of SZ patients are 10 times more likely to develop SZ than people in the general population^3,65^, and a 31% to 58% concordance rate exists in monozygotic twins^66^. Patrick *et al*. have demonstrated that genetic liability to SZ was 81% (95% confidence interval: 73%, 90%) based on results from 12 twin studies of SZ^67^. When taken with these previous results, our findings in unaffected relatives point to the possibility of altered functional interplay within DMN as the unit responsible for cerebral dysfunction and initial sign for developing SZ. Accordingly, compromised activation of the brain links with the risk of developing SZ in individuals at familial high risk.

Additionally, we performed CBF connectivity and analyzed characteristics of its network. From the perspective of sparsity, the CBF connectivity matrix of HCs was dense, but those of SZ patients and their relatives were sparse. Based on the computed global network properties^68^, including clustering index, characteristic path length, local efficiency, and global efficiency, SZ patients are unable to so efficiently transfer information within functional brain network, and they have weakened capacity against disturbance relative to HCs. Importantly, SZ patients’ relatives are with an intermediate level between HCs and patients for local efficiency and global efficiency, implying an disrupted integration in larger and sparser network^69^. Previously, Zhu *et al*. and Liu *et al*. compared CBF connectivity between SZ patients and HCs, respectively, exhibiting CBF disconnections and disrupted topological properties in CBF covariance network^10,70^. Most recently, we performed CBF connectivity analysis in SZ patients, dividing patients into groups based on symptoms^6^. However, there are not ample papers using CBF-derived connectivity to compare SZ patients and their first-degree relatives. Our study provides an initial but comprehensive view of the CBF concurrent fluctuations-based network among brain areas for SZ patients and their relatives. As well, in our previous effective connectivity study using resting-state BOLD-functional MRI, we detected an abnormal pattern of anterior cingulate cortico-hippocampal connectivity in unaffected relatives of first-episode SZ^22^. Both CBF connectivity (efficiency of blood flow distribution among brain areas) and effective connectivity (causal influence of one brain region exerting over another) provide a new perspective for understanding familial susceptibility for SZ.

However, there are some limitations in our study. First, because of the strict exclusion criteria, we recruited a relatively small sample, including 45 SZ patients, 32 unaffected relatives, and 51 HCs. This relatively small sample size may affect the statistical reliability of our results. Large sample and multi-center study is desirable to confirm our current results. We hope to enlarge the sample size in the following study, and obtain more reliable achievements in further. Second, the technique we used – ASL, a mature technique valuing CBF noninvasively – had a shortage of the fluctuation in CBF values which confronted with all research utilized this method. Compared with cerebral blood volume assessed by ASL, CBF values often tend to demonstrate different outcomes, which may affect our results. For the future study, we may utilize a combination of multimodalities, including BOLD-functional MRI, diffusion tensor imaging, magnetic resonance spectroscopy, electroencephalography, and PET, in hope of strengthening the conclusion.

In summary, the present study reveals CBF change in first-episode, drug-naïve SZ patients and their first-degree relatives, demonstrates CBF alterations pronounced in brain regions within DMN and deficit CBF connectivity patterns, and further elucidates association between CBF values with clinical symptoms. Our results may help exploring pathogenesis underlying SZ itself and the heritability in liability of SZ.

## Electronic supplementary material

Supplementary Information 
